# Evaluation of drip and flood irrigated treatments under varying heat stress on winter wheat: a four-seasons experimental study

**DOI:** 10.1038/s41598-026-36458-6

**Published:** 2026-01-20

**Authors:** Ghanshyam Giri, Hitesh Upreti, Gopal Das Singhal

**Affiliations:** 1 Water Management Field Laboratory, Department of Civil Engineering, School of Engineering, Shiv Nadar Institution of Eminence, Delhi, NCR India; 2 Department of Civil Engineering, School of Engineering, Shiv Nadar Institution of Eminence, Delhi, NCR India; 3 Department of Civil Engineering, School of Engineering, Shiv Nadar Institution of Eminence, Delhi, NCR India

**Keywords:** Winter wheat, Grain yield (GY), Water productivity (WP), Heat wave, Varying temperature, Climate sciences, Environmental sciences, Hydrology

## Abstract

Evaluating and enhancing grain yield (GY) and water productivity (WP) is crucial for ensuring food security while reducing pressure on limited water resources through more efficient water use. This is particularly important when the adverse impacts of climate change are growing, resulting in more frequent heatwaves. However, limited research has examined the response of winter wheat GY and WP to drip and flood irrigated treatments under varying heat stress induced by interannual variation in weather. Field experiments were conducted for four consecutive crop seasons [2021-22 (season 1), 2022-23 (season 2), 2023-24 (season 3), and 2024-25 (season 4)] for winter wheat. Five irrigation treatments were employed: (i) 25% MAD (maximum allowable deficit, i.e. irrigation at 25% soil moisture depletion of total available water) with drip irrigation, (ii) 50% MAD with drip irrigation, (iii) 50% MAD with flood irrigation, (iv) farmers’ field replication, and (v) rainfed treatment. Among the four seasons, the temperature and rainfall conditions varied significantly. Grain yield was analyzed using heat stress indices derived from air temperature (accumulated heat stress days, heat stress intensity & heat degree days) and observed yield (heat vulnerability index, stress tolerance index & yield stability index) to identify heat-resilient treatments that optimize water use without reducing GY. For all seasons, GY and WP values were highest for 50% MAD drip irrigation, followed by 25% MAD drip irrigation, 50% MAD flood irrigation, and farmers’ field replication. In season 1, a heatwave substantially reduced GY in farmers’ field replication, while soil moisture-based irrigated treatments showed greater resilience. In season 2, rainfall events during the grain-filling stage, in the absence of heat stress, led to the highest GY and WP in a specific treatment. In season 3, post-heading heat stress reduced grain yield across all treatments compared to season 2. In season 4, a hailstorm at the onset of grain-filling stage reduced yields across all irrigation treatments relative to seasons 2 and 3. Analysis of heat stress indices showed that the conventional irrigation method is more vulnerable to heat stress during critical growth stages, reducing GY. Soil moisture-based irrigation scheduling helps minimize the adverse impact of heat stress on wheat yield.

## Introduction

Agriculture utilizes about 70% of the freshwater consumption throughout the world^1^, while in Asia, this sector accounts for more than 80% of total freshwater withdrawals^2^. Wheat is a major crop for global food security, produced around 730 million tons annually across 2.1 million km² worldwide^3^, with an average yield of 3 tonnes/hectare^4,5^. As documented by the World Bank, wheat production must grow by 60% to meet the food demands of a global population projected to reach 9.6 billion by 2050^6^. However, global water availability is under threat due to climate change and population growth^7^. Consequently, prioritizing sustainable water use in agriculture enhances water use efficiency (WUE) and helps preserve limited resources^8,9^. WUE in developing countries are extremely low^10,11^. The deficit irrigation concept can enhance WUE in agriculture^12^. When considering deficit irrigation, it is essential to ensure that it does not adversely impact crop productivity, as providing food security for the growing population remains a crucial priority.

Enhancing irrigation water productivity (IWP) is vital for ensuring food security and reducing the strain on limited water resources by optimizing their use^13^. IWP is defined as the ratio of marketable crop yield to applied irrigation water. IWP measures the efficiency of utilizing irrigation water for agricultural purposes by quantifying crop yield relative to the water applied^14^, making it a key indicator of agricultural output and WUE. It also serves as a comprehensive indicator to measure the ability of irrigation systems and crop management practices^15,16^. In addition to IWP, crop water productivity (CWP) also stands as a significant indicator, offering insights into regional agricultural production and serving as a measure of food sustainability^17^. CWP, defined as the ratio of marketable crop yield to water consumed through evapotranspiration^18^, is a valuable tool for evaluating irrigation scheduling, particularly under deficit irrigation conditions^19^. It measures the efficiency of agricultural water use^13,20,21^. Therefore, obtaining quantitative information on IWP and CWP becomes very important for planning irrigation water management in a region. Water productivity can be improved by reducing the irrigation water use. Irrigation treatments with variations in deficit serve as a valuable approach in reducing irrigation water use^22,23^. Effective irrigation management improves crop water productivity (WP) by regulating the timing and application of irrigation, delivering only the necessary water for crops. For these reasons, interest in drip irrigation is growing in water-scarce regions^24^. Drip irrigation conserves water and reduces energy consumption because the system operates at low pressure. Targeted application of water at the plant roots promotes healthier growth and reduces the risk of diseases. Additionally, since drip irrigation minimizes surface flooding and standing water, it effectively suppresses weed growth. Several studies have explored the impact of different irrigation treatments on crop yield and water productivity for winter wheat^24–35^. In various studies, irrigation was altered by reducing the water application in one or more crop phenological stages^28,36^. Some of these studies varied irrigation amounts based on crop water/irrigation requirements^29,37,38^. A few studies altered irrigation based on the ratio of irrigation water to cumulative pan evaporation (IW: CPE)^35^ and based on soil moisture deficit (SMD) from field capacity^24,31^.

Further, climate change affects crop yield, which can affect both IWP and CWP. Climate change has varying effects on crop yields, with positive impacts observed in certain agricultural regions and negative impacts in others^39,40^. As per the latest document from the FAO (Food and Agriculture Organization of the United Nations), under high-emission climate scenarios, there could be a decline in production ranging from 20 to 45% for maize, 5–50% for wheat, 20–30% for rice, and 30–60% for soybeans due to climate change by 2100^41^. There is a prediction of decrease in cereal crop yield of 4% to 10% in southern Asia by the 21 st century^42^. Variations in temperature, precipitation, and other climatic elements affect the availability of light, heat, and water essential for crop growth, subsequently affecting crop physiology, yield, and quality. While the degree of influence varies by crop type, the overall impact is expected to be substantial^43,44^. Recent studies project a significant rise in the occurrence of drought and heat stress during the anthesis and grain-filling stages of wheat by the end of the 21 st century due to climate change^45,46^. Moreover, exposure to hot and dry winds during the grain-filling period has been associated with accelerated senescence in winter wheat, leading to reduced grain yield^47^. Alterations in precipitation patterns, such as high-intensity rainfall, are becoming more common and can severely impact crop production, especially during sensitive stages^48^. Hailstorms also significantly impact agricultural productivity by leading to considerable reductions in crop yield and interfering with routine farm management practices^49^. Additionally, hail-induced injuries on plant surfaces can serve as entry points for various pathogens, thereby exacerbating yield losses and compromising crop quality^50,51^. Hence, there is a need to conduct quantitative assessments of how crop yields respond to variable climate. A study^52^ investigated the impact of post-flowering drought and supplemental irrigation on wheat yield by comparing rainfed conditions across multiple wheat genotypes. Various studies^53–55^ have demonstrated the effect of climate change on the GY of winter wheat. These studies primarily employed yield simulation models to analyze trends under varying climatic conditions. Additionally, the implications of increasing and fluctuating temperatures on wheat productivity have been explored in the literature^56–59^, wherein modelling approaches were utilized to quantify temperature-induced yield responses. Moreover, combined effects of temporal trends in temperature and water-related variables on wheat yield outcomes using modeling approach was investigated^60^. In these studies, crop yield was simulated by manipulating the temperature stress conditions using the models.

Extensive research has been conducted on impacts of climate change on winter wheat through modelling approach by manipulating or creating heat stress conditions using the models. However, there remains a critical need for field-based experimental studies that have quantified the effect of varying levels of heat stress and rainfall induced by interannual variation in weather during the multiple crop seasons. This is particularly important for evaluating yield responses under different irrigation methods and treatments. This study investigates the inter-seasonal variability in yield and water productivity (IWP and CWP) of winter wheat in response to interannual fluctuations in heat stress and rainfall over four consecutive cropping seasons. The analysis considers drip and flood irrigation treatments under varying soil moisture levels, conventional irrigation practices, and rainfed conditions within a humid subtropical climate. This study also utilizes heat stress indices based on temperature and yield data to observe the effect of heat stress on wheat yield.

## Materials and methods

### *Description of the experimental site*

**Fig. 1:**
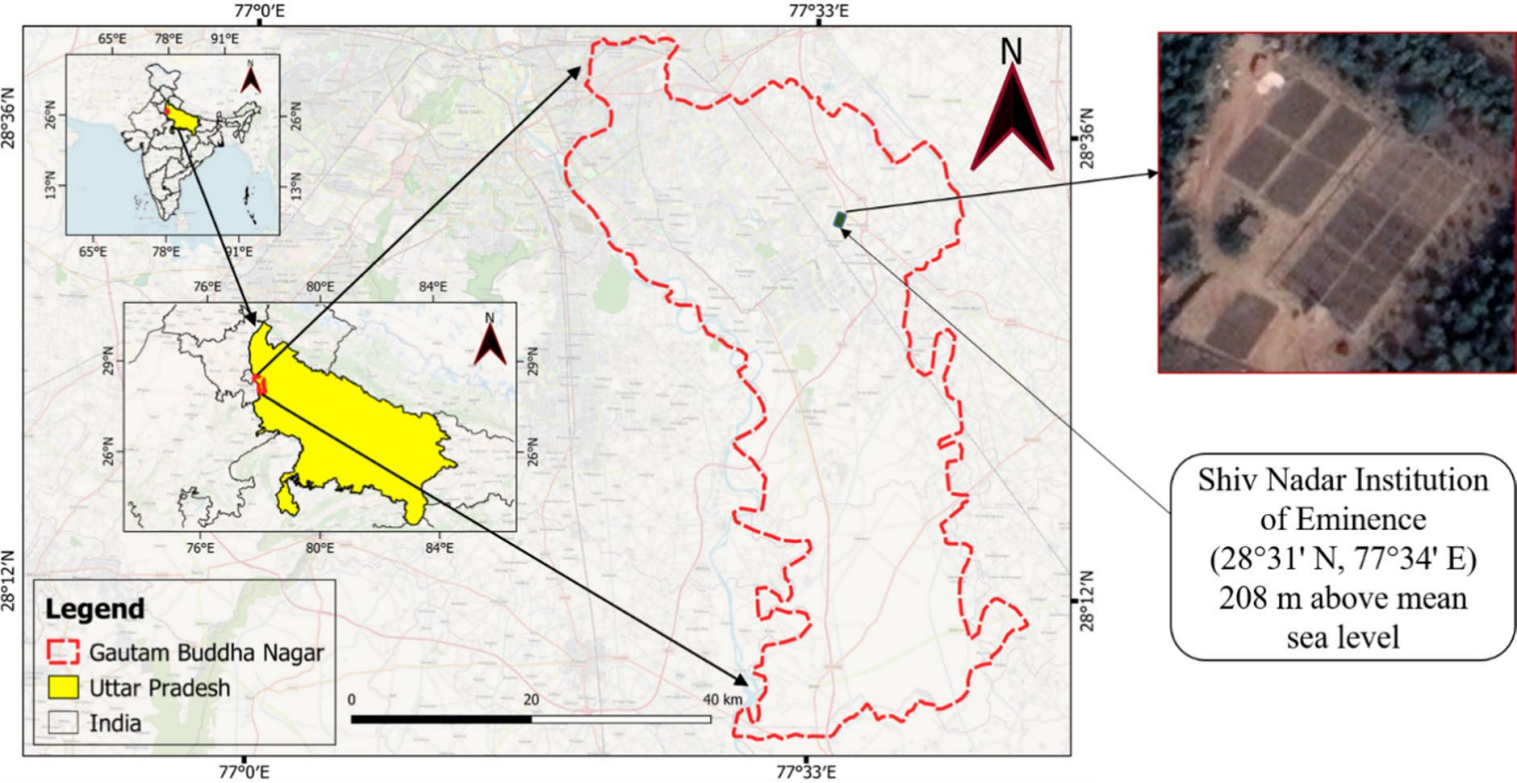
Location of the experimental site (Fig. 1 was created using QGIS version 3.22.15 (https://download.qgis.org/downloads/). The Google Earth Pro imagery dated 12 February 2022 was used to display the experimental plots in the Figure.

Field experiments were conducted during four consecutive wheat crop seasons (2021-22, 2022-23, 2023-24, and 2024-25) at the Water Management Field Laboratory, Shiv Nadar Institution of Eminence, Uttar Pradesh. The location of the experimental site is shown in Fig. 1. Hereafter, the wheat crop season 2021−22 will be referred to as season 1, the 2022-23 season as season 2, the 2023-24 season as season 3, and the 2024-25 season as season 4. The climate of the study area is classified as humid subtropical (Cwa) according to the Koppen-Gieger climate classification system^61,62^. The soil in the study area is of the sandy loam type.

Before sowing, the field was prepared for conducting experiments by ploughing and levelling. The wheat variety DBW173 was sown manually on 16 December 2021 during season 1, 5 December 2022 during season 2, 12 December 2023 during season 3, while the date of sowing was 30 November 2024 during season 4. The wheat variety DBW 173 was procured from the ICAR–Indian Institute of Wheat and Barley Research (IIWBR), Karnal, Haryana, India (seasons 1 and 2) and from Sardar Vallabhbhai Patel University of Agriculture and Technology, Meerut, Uttar Pradesh, India (seasons 3 and 4). This genotype (DBW173) is heat tolerant and recommended for irrigated late-sown conditions. This genotype is also resistant to yellow and brown rust (disease resistant). The recommended regions for this genotype are North Western Plains Zone (NWPZ) of India, including parts of Punjab, Haryana, Delhi, Rajasthan, Western UP, Jammu & Kashmir, Himachal Pradesh (Una district and Paonta valley), and Uttarakhand (Tarai region). The sowing rate remained consistent at 40 kg/ha across all four seasons. Row-to-row spacing was maintained at 20 cm, with 10 cm spacing between plants within rows. The plots were kept weed-free by removing weeds manually at regular intervals throughout each crop period in all the seasons. The crop was harvested on 14 April 2022 in season 1, 8 April 2023 in season 2, 15 and 16 April 2024 in season 3, while the crop was harvested on 6 and 7 April 2025 in season 4.

### Weather data

The weather data for all four crop seasons (2021-25) was collected from an automatic weather station (AWS) inside the study area. The monthly averaged climatic parameters (maximum and minimum air temperature (T_max,_ and T_min_), maximum and minimum relative humidity (RH_max_ and RH_min_), wind speed (u), incoming radiation (R_S_) and rainfall) during the crop period across four seasons are tabulated in Table 1.

**Table 1:** Variation of monthly-averaged meteorological parameters during the four crop seasons.

Experimental Years	Climatic Parameters	Dec	Jan	Feb	Mar	Apr
Season 1 2021–2022	T_max_ (°C)	20.9	19.5	25.2	33.2	39.7
	T_min_ (°C)	6.2	9.0	9.9	16.0	18.4
	RH_max_ (%)	99.3	94.9	92.8	89.2	69.5
	RH_min_ (%)	46.0	66.2	44.5	32.6	12.6
	u (m/s)	0.3	0.4	0.6	0.5	0.5
	R_S_ (MJ day^− 1^ m^− 2^)	9.8	7.7	13.7	16.5	18.1
	P (mm)	0	82.30	18.20	0	0
Season 2 2022–2023	T_max_ (°C)	22.4	18.4	26.7	28.9	30.8
	T_min_ (°C)	8.8	7.5	11.5	15.3	16.1
	RH_max_ (%)	94.4	95.0	91.6	94.0	88.4
	RH_min_ (%)	47.7	59.7	41.9	47.6	37.5
	u (m/s)	0.4	0.4	0.5	0.4	0.5
	R_s_ (MJ day^− 1^ m^− 2^)	8.1	7.4	12.5	13.4	17.83
	P (mm)	46.7	29.1	0	72.8	1.6
Season 3 2023–2024	T_max_ (°C)	21.3	15.6	23.3	28.6	35.4
	T_min_ (°C)	8.6	6.9	10.2	14.5	18.2
	RH_max_ (%)	96.6	97.1	91.3	86.8	69.6
	RH_min_ (%)	56.3	72.6	44.4	33.1	20.1
	u (m/s)	0.5	0.5	0.6	0.6	0.6
	R_s_ (MJ day^− 1^ m^− 2^)	9.3	5.9	10.6	13.7	14.9
	P (mm)	0	18.1	11.40	4.80	0.0
Season 4 2024–2025	T_max_ (°C)	21.8	19.6	25.1	30.6	36.7
	T_min_ (°C)	8.6	8.7	11.7	14.5	18.2
	RH_max_ (%)	95.4	94.1	90.1	84.2	68.7
	RH_min_ (%)	56.0	69.3	42.1	31.6	18.5
	u (m/s)	0.4	0.5	0.6	0.6	0.6
	R_s_ (MJ day^− 1^ m^− 2^)	9.5	6.2	11.4	14.5	15.4
	P (mm)	17.2	16.3	1.6	9.8	0.0

### Irrigation treatments

The experiment consisted of five irrigation treatments across all four seasons. In each of the four seasons, every treatment was replicated three times, with each replication measuring 5 m × 4 m. The replications of all irrigation treatments were arranged in a split-plot design during all the seasons. In these treatments, volumetric soil moisture values (cm^3^ cm^− 3^) were measured three times a week during the entire crop period, using a Delta-T profile probe meter (PR2/6) manufactured by Delta-T Devices Ltd., Cambridge, UK. Before sowing the wheat crop in season 1, the field capacity (FC) values were estimated using an experimental procedure^63^. According to this procedure, the plots were irrigated such that the soil was saturated. Then, the soil surface was covered with plastic covers to avoid water loss due to evaporation. After this, the soil moisture values were measured twice every 24 h. When the depletion in soil moisture became negligible, the values were considered FC. The average FC values with soil texture and bulk density are listed in Table 2. Average organic carbon content of 0–15 cm depth was 0.65%. The permanent wilting point value was taken as 6.8% v/v for sandy-loam soil^64^. Out of five, three irrigation treatments were based on the maximum allowable deficit (MAD). The MAD is a key irrigation management threshold that defines the maximum proportion of soil water a crop can deplete before experiencing water stress. Expressed as a percentage of total available water in the root zone, MAD guides the amount of irrigation required to restore soil moisture to field capacity. The MAD was estimated using Eq. (1)^65^.

1 $$\:MAD=\frac{1}{n}\sum\:_{i=1}^{n}\frac{F{C}_{i}-{\theta\:}_{i}}{{FC}_{i}-PWP}\times\:100$$

where n is the number of layers (*n* = 4 for the present study, the depth of each layer considered is 10 cm), $\:F{C}_{i}$ is the field capacity (%) of the i^th^ layer, $\:{\theta\:}_{i}$ is the soil moisture content of the i^th^ layer, and $\:PWP$ is the permanent wilting point (%).

**Table 2:** Field capacity (FC), soil texture, and bulk density values for all five irrigation treatments.

Soil Depth (cm)	0–10	10–20	20–30	30–40	40–50	50–60
FC (%)	24.5	26.6	29.3	31.7	34.3	37.2
Soil Texture	Sandy loam	Sandy loam	Sandy loam	Sandy loam	Sandy loam	Sandy loam
Bulk Density (g cm^− 3^)	1.47	1.50	1.52	1.54	1.58	1.61

In the first irrigation treatment, the plot was irrigated every time the soil moisture in the plot depleted to 25% of the total available water (TAW). TAW is difference between the values of soil moisture at FC and PWP. This plot, equipped with a drip irrigation system, was referred to as a ʺfully irrigated dripʺ plot. In the drip irrigation system, drip laterals were spaced 40 cm apart, with each lateral containing 14 emitters. Each emitter had a discharge rate of 2.3 L per hour. In the second irrigation treatment, the plot was irrigated whenever soil moisture dropped to 50% of TAW. This plot, also utilizing a drip irrigation system, was referred to as ʺ50% MAD dripʺ plot. The third irrigation treatment was similar to the second, with irrigation occurring when soil moisture reached 50% of TAW. This treatment used a flood irrigation system and was referred to as ʺ50% MAD floodʺ plot. The 50% threshold serves as a benchmark for managing irrigation in wheat, helping to maintain adequate soil moisture for crop growth while minimizing unnecessary water use^66^. The 25% threshold was considered to maintain the soil moisture near FC during the entire crop period. The timing and quantity of irrigation in these three treatments varied, as they were determined by the soil moisture content (SMC) in the root zone. The irrigation scheduling in this study considered an effective root zone depth of 40 cm based on previous research conducted in similar climates and soil types^67,68^. In the fourth irrigation treatment, irrigation followed the prevailing thumb rules used by the farmers in the local area, irrigating wheat 4–5 times at 20–25-day intervals throughout the crop period. This treatment was facilitated with a flood irrigation system and referred to as a ʺfarmers’ field replicationʺ. In the fifth irrigation treatment, water was applied only once during the crown root initiation (CRI) stage using flood irrigation as a life-saving irrigation. Later, rainfall served as the irrigation source for the remainder of the crop period. This treatment was termed ʺrainfedʺ. The details of the irrigation treatments are tabulated in Table 3.

**Table 3:** Details of irrigation treatments.

Irrigation treatment	Plot No.	Maximum Allowable Deficit (MAD)	Types of irrigation
Fully Irrigated Drip	Plot 1	25%	Drip
50% MAD Drip	Plot 2	50%	Drip
50% MAD Flood	Plot 3	50%	Flood
Farmers’ Field Replication	Plot 4	-	Flood
Rainfed	Plot 5	-	-

### Estimation of heat stress indices

In the present study, for the evaluation of the grain yield of the irrigation treatments under varying levels of heat stress, different temperature indices were estimated, as mentioned in Table 4. These indices were derived from temperature data, using 30 °C as the threshold for defining heat stress^69,70^.

**Table 4:** Heat stress indices based on temperature data

Index	Abbreviation	Unit	Definition
Accumulated heat stress days	AHSD	days	Number of days with T_max_ ≥ T_h_ during the post-heading period
Heat stress intensity	HSI	^0^C	Average T_max_ on days with T_max_ ≥ T_h_ during the post-heading period
Heat degree days	HDD	^0^C day	Total post-heading heat degree-days, estimated using Equations (2) and (3)

T_max_: daily maximum temperature, T_h_: the threshold temperature for heat stress, fixed as 30 °C.

2 $$\:HDD={\sum\:}_{{d}_{h}}^{{d}_{m}}{HD}_{i}$$

3 $$\:{HD}_{i}=\left\{\begin{array}{c}\:0,\:\:{T}_{maxi}<{T}_{h}\\\:{T}_{maxi}-{T}_{h},\:\:{T}_{maxi}\ge\:{T}_{h}\end{array}\right.$$

In Eqs. (2) and (3), $\:{d}_{h}$ and $\:{d}_{m}$ represent the heading and maturity dates, while $\:{T}_{maxi}$ is the daily maximum temperature on day *i* and $\:{T}_{h}$ is the threshold temperature for heat stress. $\:{HD}_{i}\:$is the heat degree days above the 30 °C threshold on day *i*. $\:HDD$ is the cumulative sum of $\:{HD}_{i}$ from heading to maturity.

In addition to temperature-based heat stress indices, the heat vulnerability index (HVI)^71^, stress tolerance index (STI)^72^ and yield stability index (YSI)^73^ were calculated using grain yield data through Eqs. (4), (5), and (6), respectively. These indices were computed to find out the heat-tolerant irrigation treatments.

4 $${\rm Heat \:Vulnerability \:Index\: (HVI)};\:\:\:\:\:HVI=\:\frac{\left(1-\frac{{GY}_{S}}{{GY}_{P}}\right)}{\left(1-\frac{{AGY}_{S}}{{AGY}_{P}}\right)}$$

5 $${\rm Stress \:Tolerance \:Index\: (STI);}\:\:\:\:STI=\frac{{GY}_{P}\times\:{GY}_{S}}{\:{{AGY}_{P}}^{2}}$$

6 $${\rm Yield \:Stability \:Index \:(YSI);}\:\:\:\:YSI=\:\frac{{GY}_{S}}{{GY}_{P}}$$

Where, $\:{GY}_{P}$ and $\:{GY}_{S}\:$represent the grain yield of treatments under normal and heat stress conditions, respectively. Whereas $\:{AGY}_{P}$ and $\:{AGY}_{S}$ are the average yields of all treatments under normal and heat stress conditions, respectively. The grain yield of all the treatments was considered to be in normal condition during season 2 (explained in Sect. 3.1).

### Computation of irrigation water productivity (IWP) and crop water productivity (CWP)

The wheat crop was manually harvested for each plot separately, and grain yield (GY) was recorded using a weighing machine in kg/ha. In the present study, IWP was computed as the ratio of GY obtained to the amount of irrigation applied to the field (Eq. (7)). The estimation of the irrigation amount applied to the field is explained in Sect. 2.5.1. Further, the CWP was estimated as the ratio of GY obtained to the amount of water consumed through crop evapotranspiration (ET_C_) (Eq. (8)). To calculate CWP, ET_C_ was determined using the FWB method (Sect. 2.5.2).

7 $$\:IWP=\:\frac{Grain\:Yield\:(kg/ha\:)}{Irrigation\:amount\:applied\:\left(mm\right)}$$

8 $$\:CWP=\:\frac{Grain\:Yield\:(kg/ha)}{Crop\:Evapotranspiration\:\left(mm\right)}$$

Here, $\:IWP$ and represent irrigation water productivity and crop water productivity, respectively.

#### Amount of irrigation applied

In a drip and flood irrigation system, the amount (in mm) of irrigation applied was estimated using Eq. (9).

9 $$\:{I}_{A}={\sum\:}_{i=1}^{4}({PWP+TAW)}_{i}-{\theta\:}_{i})$$

Here $\:{I}_{A}\:$is the amount of irrigation (mm) applied through a drip or flood irrigation system, $\:PWP$ represents the permanent wilting point (mm),$\:\:TAW$ is the total available water (mm), and the subscript *i* represents the *i*^th^ layer.

#### Field water balance (FWB)

Crop evapotranspiration (*ET*_*C*_) was calculated as the residual of the FWB using Eq. (10).

10 $$\:{ET}_{C}=\left(RF+I\right)\pm\:\varDelta\:S-DP-RO\pm\:\varDelta\:HF-CR$$

where $\:{ET}_{C}$ is the crop evapotranspiration (mm); $\:RF$ is the amount of rainfall (mm); $\:I$ is the amount of irrigation applied (mm); $\:\varDelta\:S$ is the change in storage (mm); $\:DP$ is deep percolation below the root zone (mm); $\:RO$ is surface runoff (mm);$\:\:\varDelta\:HF$ is horizontal flow in or out of the root zone below the surface and $\:CR$ is capillary rise. $\:\varDelta\:S$ represents the soil moisture storage during the crop season. In the present study, $\:RO$ was taken as zero since no surface runoff occurred during the crop period. Also, $\:\:\varDelta\:HF\:$It was assumed to be negligible since each subplot was separated from the others. Further, $\:CR$ was taken as zero since the water table depth in the study area was 1.5 m below the surface. Finally, Eq. (10) was reduced to Eq. (11):

11 $$\:{ET}_{C}=\left(RF+I\right)\pm\:\varDelta\:S-DP$$

The deep percolation in the study was estimated based on the deficit and the irrigation applied to plots. For this purpose, the deficit on$\:\:\:t1$^*th*^ day was estimated through Eq. (12).

12 $$\:{Deficit}_{t1}=\sum\:_{i=1}^{4}({FC}_{i}-{\theta\:}_{it1})$$

Where $\:{FC}_{i}$ is the field capacity of the i^th^ layer, and $\:{\theta\:}_{it1}\:$is the volumetric soil moisture content of the i^th^ layer on $\:t1$^*th*^ day. If the amount of rainfall/irrigation (RF/I) on $\:t1$^*th*^ day exceeded the deficit of that day, the surplus amount was taken as DP. Otherwise, DP was considered as zero. Here, $\:t1$^*th*^ day is the day when either rainfall occurred, or irrigation was applied.

### Statistical analysis

A one-way ANOVA was conducted to evaluate whether the mean GY, IWP, CWP, varied significantly among the irrigation treatments across four seasons. To identify specific treatment pairs with significant differences, Tukey’s HSD test was applied as a post hoc analysis. Also, one-way ANOVA test was applied to check whether rainfall, and maximum temperature varied significantly across the four seasons. All statistical tests were conducted at a 95% confidence interval.

## Results and discussion

### Variation in temperature and rainfall patterns across four seasons

**Fig. 2:**
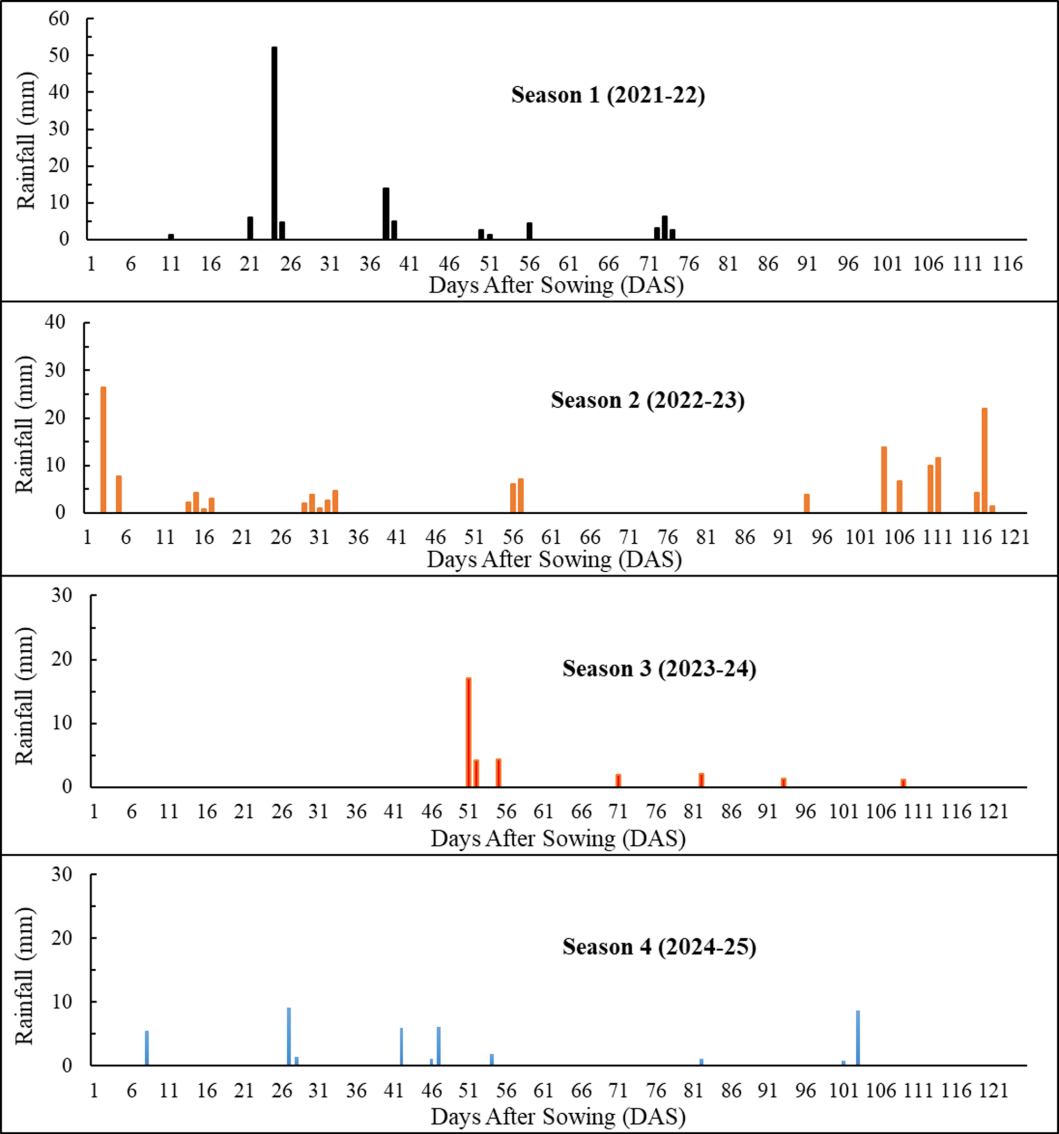
Daily rainfall during the four wheat crop seasons (p-value < 0.05**)**.

**Fig. 3:**
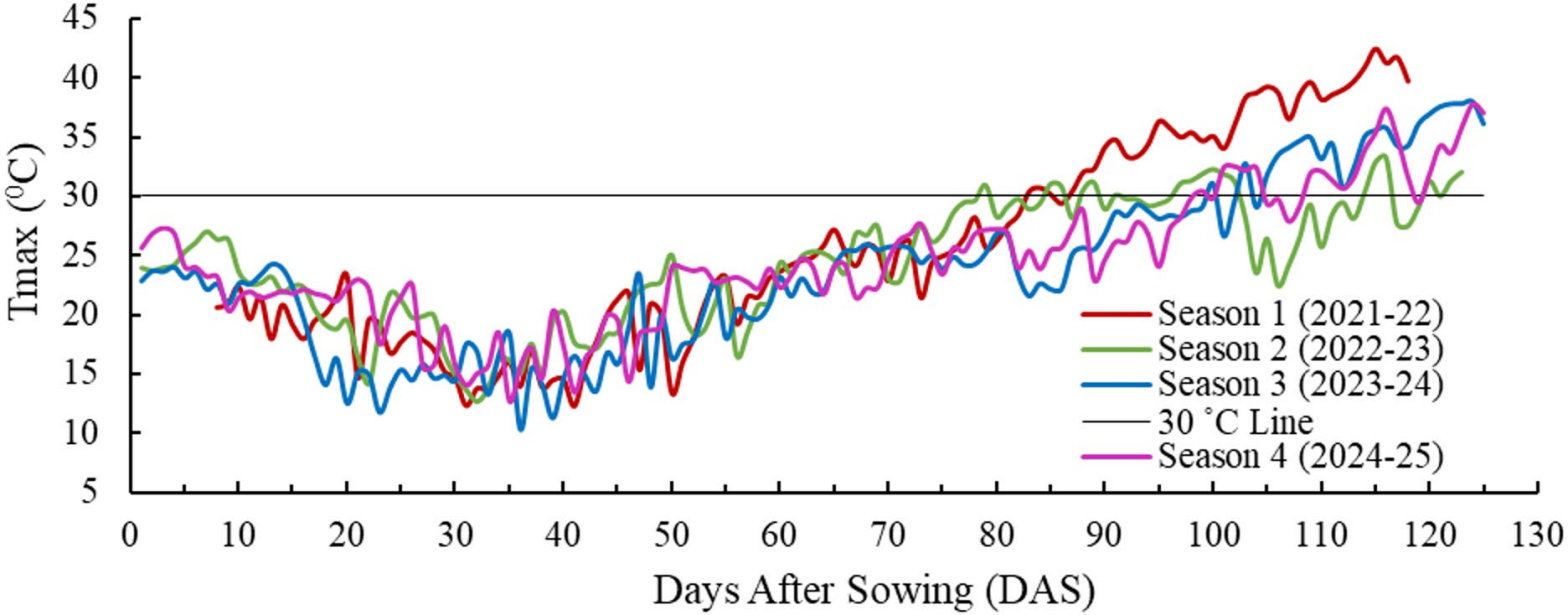
Time series of maximum air temperature (T_max_) for the four crop seasons (p-value < 0.03**).**

During the four crop seasons studied, the temperature and rainfall patterns varied significantly. The time series of daily rainfall and daily maximum air temperature (T_max_) for the four seasons are shown in Figs. 2 and 3, respectively. In season 1, a significant event of 52.1 mm occurred during the crown root initiation (CRI) stage. However, there was no rainfall during the flowering and grain-filling stages. In this season, the T_max_ varied between 12.35 °C and 42.46 °C, with the crop experiencing a heat wave during the grain-filling stage^74^. In season 2, only a minimal amount of rainfall (0.3 mm) occurred during the CRI stage, while 19.4 mm occurred during the active tillering stage. However, 3.9 mm and 20.7 mm of rainfall were recorded during the flowering and grain filling stages, respectively. The temperature during the later part of the crop season was lowest in this season, and T_max_ varied between 12.72 °C and 33.24 °C. Season 3 experienced the lowest rainfall of the four seasons. During this season, 0.2 mm and 26.6 mm of rainfall occurred at the CRI and jointing stages, respectively. There was also 1.4 mm of rainfall during the flowering stage and only 0.1 mm during the grain-filling stage. In this season, T_max_ varied between 10.3 °C and 38 °C. In Season 4, 10.9 mm of rainfall was recorded during the CRI stage, followed by 1.5 mm during the flowering stage. During the beginning of the grain-filling stage, a total of 9.7 mm of rainfall occurred, which included a severe hailstorm event contributing 8.7 mm. The heat stress indices based on temperature data across four seasons are tabulated in Table 5. The threshold temperature for estimation of these indices was considered as 30 °C^69,70^. Among the four seasons, all heat stress indices recorded the highest values in season 1, followed by seasons 4 and 3, with the lowest values observed in season 2. In contrast, post-heading rainfall was highest in season 2 and lowest in season 1. Accordingly, season 2 was assumed to be the least heat-stressed season (normal season) in this study, while the remaining seasons were identified as being affected by heat stress during the post-heading period.

**Table 5:** Heat stress indices (post-heading to maturity) based on temperature data across four seasons.

Index	Unit	Season 1 (2020-21)	Season 2 (2021-22)	Season 3 (2023-24)	Season 4 (2024-25)
Accumulated heat stress days (AHSD)	Days	35	15	23	26
Heat stress intensity (HSI)	^0^C	36.3	31.3	34.1	34.9
Heat degree-days index (HDD)	^0^C days	220.5	25	103.6	110.2

### Evaluation of grain yield for crop seasons with varying temperature and rainfall patterns for drip and flood irrigated treatments

**Fig. 4:**
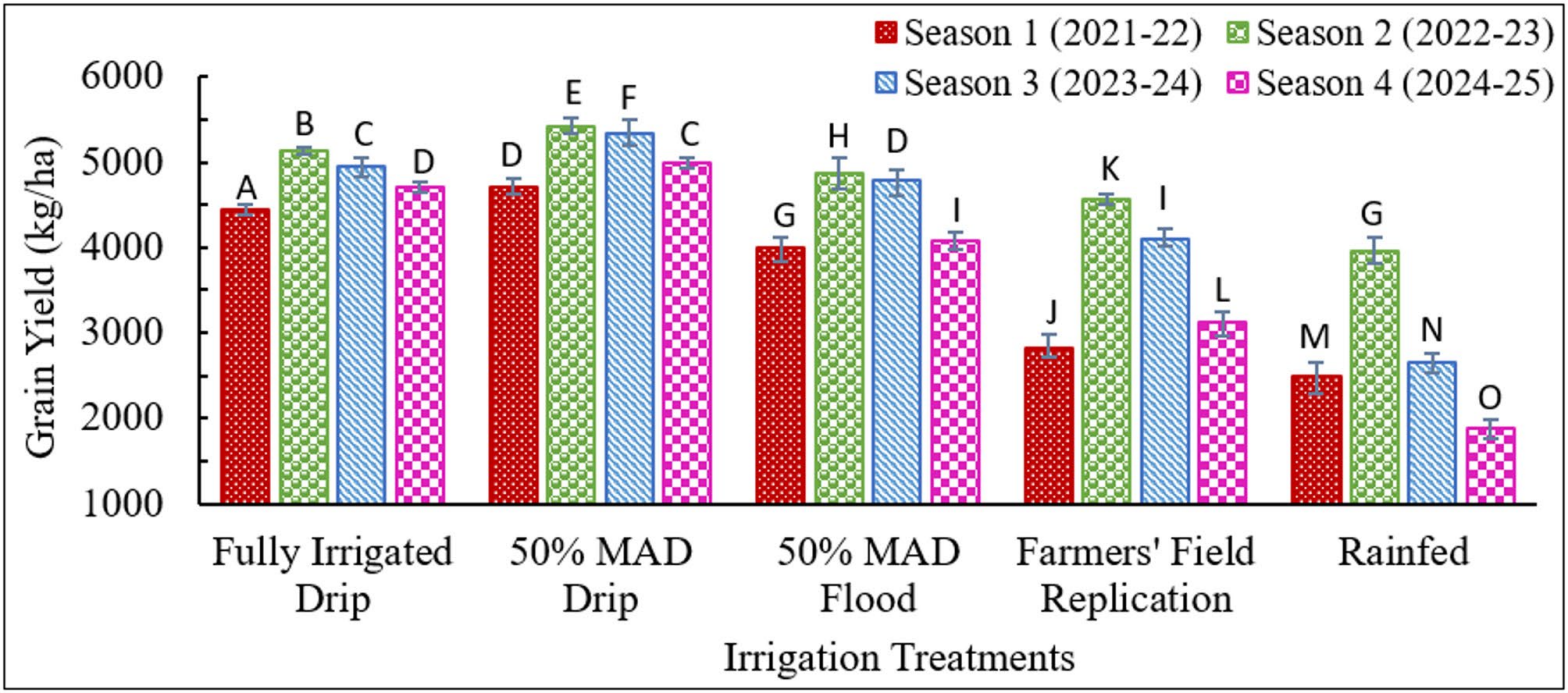
Average observed grain yield for different irrigation treatments in the four crop seasons. (MAD is maximum allowable deficit; Alphabets in the Figure shows statistical difference; same alphabets show statistical similarity in the values)

The average observed GY for all five irrigation treatments across all four seasons is shown in Fig. 4. The error bars show the maximum and minimum GY in each treatment across the seasons. One-way Analysis of Variance (ANOVA) was performed to assess whether the mean yields differed significantly among the irrigation treatments. Subsequently, Tukey’s Honestly Significant Difference (HSD) test was employed as a post hoc procedure to determine which specific pairs of treatments showed statistically significant differences in yield. GY exhibited significant differences among the treatments, with p-values less than 0.05. Within individual seasons, the GY of all treatments differed from others, showing statistical significance at *p* < 0.01. In the fully irrigated drip, farmers’ field replication, and rainfed treatments, inter-seasonal variations in GY were statistically significant, with p-values below 0.02. For the 50% MAD drip and 50% MAD flood treatments, seasonal differences in GY were also significant, with p-values ranging from 0.03 to 0.05.

**Fig. 5:**
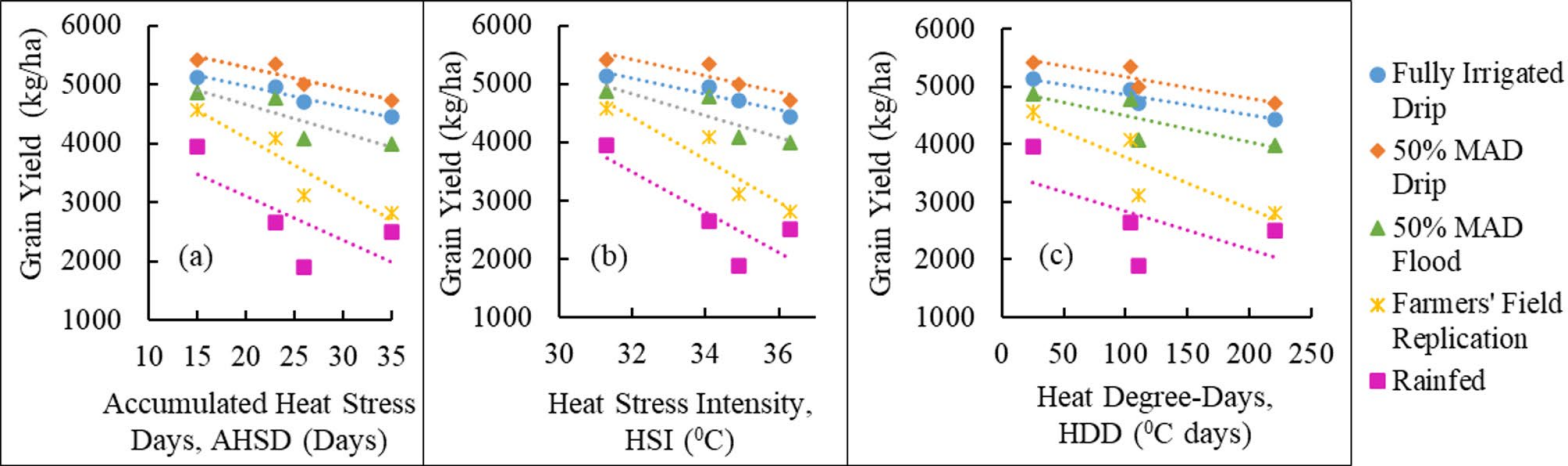
Relationship between grain yield (GY) and heat stress indices based on temperature data *(MAD is maximum allowable deficit)*.

In all the seasons, the GY was highest for the 50% MAD drip, followed by fully irrigated drip, 50% MAD flood, farmers’ field replication, and rainfed treatment. Comparing the four seasons, the GY of a specific irrigation treatment was highest in season 2, followed by season 3, season 4, and lowest in season 1, except in rainfed. The reason for the lowest GY for all the treatments in season 1 can be attributed to the occurrence of the heat wave in the study region during March 2022, which coincides with the grain-filling stage of the crop season^74^. This can also be clearly seen in the daily maximum temperature values of season 1 (Fig. 3). The study^74^ reported that the heat wave coincided with the later stages of wheat, causing yellowing, shrivelling of grain, and forced maturity, resulting in a 15–25% yield reduction in north India. According to^75^, the 2022 heatwave in India affected major wheat-growing areas in the northwestern and central regions of the country, resulting in yield losses of up to 15%. Dry and hot winds occurring during the grain-filling stage have been reported to accelerate senescence in winter wheat, leading to a reduction in grain yield^47^. In season 2, rainfall events during the grain-filling stage in this season (Fig. 2) ensured good water availability in the water-sensitive stage. Being the normal season in terms of heat stress and the occurrence of rain events in the grain-filling stage resulted in the highest GY for all the treatments in season 2 compared with the corresponding treatments in the other three seasons. Increased occurrences of intense rainfall within specific stages could influence crop production, particularly if these coincide with sensitive stages of the crop period was also reported in literature^48^. Seasons 3 and 4 exhibited similar temperature and rainfall patterns, with the primary distinction being the occurrence of a hailstorm during the post-heading stage in season 4. In season 4, the occurrence of a hailstorm during the beginning of the grain-filling stage reduced the GY of all irrigation treatments compared to seasons 2 and 3. Other studies^49,50^ have also reported that hailstorms adversely affect agriculture by causing substantial yield losses and disrupting farming operations and management practices. Further, GY of all irrigation treatments was analyzed in relation to the AHSD, HSI, and HDD indices. The relationships between GY and these heat stress indices are illustrated in Figs. 5(a), 5(b), and 5(c), respectively. As the values of these indices increased, the values of GY decreased for each irrigation treatment. The rate of decrease in GY in farmers’ field replication and rainfed treatments was higher than in other treatments, with an increase in these indices.

Further, the GY of the farmers’ field replication was compared with the irrigation treatments based on MAD (both drip and flood irrigated) for all four seasons. Compared to the farmers’ field replication treatment, the GY of fully irrigated drip increased by 57.9%, 12.3%, 21%, and 50.8% in seasons 1, 2, 3 and 4, respectively. For the 50% MAD drip treatment, the GY increased by 67.6%, 18.8%, 30.5%, and 60.1% in seasons 1, 2, 3, and 4, respectively. Similarly, for the 50% MAD flood treatment, the GY increased by 41.7%, 6.7%, 16.9%, and 30.6% in seasons 1, 2, 3, and 4, respectively. This shows that the grain yield of the farmers’ field replication was severely affected in season 1 due to the heat wave in that season. This is because this treatment was irrigated based on the irrigation thumb rules followed by the local farmers, unlike the other irrigated treatments in which irrigation was supplied at the pre-decided soil moisture depletion levels. Also, this treatment was severely affected by the hailstorm in season 4. Furthermore, the GY for all irrigation treatments was averaged for four seasons. The four seasons’ averaged GY for fully irrigated drip, 50% MAD drip, 50% MAD flood, farmers’ field replication, and rainfed treatments were 4804.4, 5116.4, 4427.9, 3646.1, and 2746.4 kg/ha, respectively. Overall, a higher GY was found in drip-irrigated treatments compared to flood-irrigated treatments. For the same level of irrigation application (50% MAD), the GY in drip irrigated treatment increased by 11.3% to 18.2% across the seasons, compared to flood irrigated treatment. A higher GY in drip irrigated treatments than flood irrigated treatments have been reported in the literature^35^. The study^35^ reported an increase in GY of drip irrigated treatment by 8.4% to 15.3% compared to flood irrigated treatment for the same level of irrigation (50% MAD). A few studies^24,75^ reported that increased yield under a drip irrigation system could be attributed to the greater and consistent availability of soil moisture and nutrients.

### Stable and vulnerable irrigation treatments under varying heat stress conditions

Based on the observed GY, the HVI, STI, and YSI indices for seasons 1, 3, and 4 were calculated to find out the stable and vulnerable irrigation treatments to mitigate the effect of extreme temperature conditions that occur during the critical growth stage of the wheat crop. The values of these indices for all irrigation treatments for seasons 1, 3, and 4 are tabulated in Table 6. The values of HVI were higher in the farmers’ field replication across the seasons. This shows that these respective treatments are more vulnerable to heat stress and hailstorms, compared to other treatments. Further, the values of STI and YSI were higher in scientifically managed irrigation treatments (fully irrigated drip, 50% MAD drip, and 50% MAD flood). This shows that scientifically managed treatments are more stable and heat-tolerant compared to conventional treatment (farmers’ field replication). Analysis of GY across four seasons demonstrated that scientifically managed irrigation treatments effectively alleviated the impact of heat stress or heat waves during the critical growth stage, i.e., the grain-filling stage, during season 1. In contrast, the conventional treatment was most affected by the heat wave in season 1. This is attributed to the fact that, unlike the scientific treatments, which were based on specified soil moisture deficits, the farmers’ field replication experienced higher levels of soil moisture deficit due to thumb rule-based water applications. In season 2, significant rainfall during the grain-filling stage mitigated this, and thus, the difference between the grain yields of the farmer’s field replication and scientifically managed irrigation treatments was the least. In season 4, a hailstorm during the grain-filling stage affected the conventional treatment the most. Numerous studies^77–80^ have examined the influence of heat stress on various winter wheat genotypes using GY-based heat stress indices. These studies typically treated timely sown genotypes as representing optimal growing conditions, whereas late-sown genotypes were exposed to heat stress conditions. The studies using these heat stress indices for finding the heat-tolerant irrigation treatment are scarce.

The results suggest that the farmers’ field replication (conventional agricultural practice) and rainfed treatment are more susceptible to heat waves or stress during critical growth stages, leading to a significant reduction in GY. The impacts of the varying levels of heat stress on wheat yield can be mitigated by using scientific irrigation treatments in place of prevailing irrigation thumb rules.

**Table 6:** Heat stress indices based on observed grain yield for all irrigation treatments across three seasons (1, 3, and 4) (MAD is maximum allowable deficit).

	Heat Vulnerability Index (HVI)			Stress Tolerance Index (STI)			Yield Stability Index (YSI)		
Treatments	Season 1	Season 3	Season 4	Season 1	Season 3	Season 4	Season 1	Season 3	Season 4
Fully Irrigated Drip	0.44	0.20	0.28	0.82	0.91	0.87	0.87	0.96	0.92
50% MAD Drip	0.43	0.08	0.27	0.92	1.04	0.97	0.87	0.98	0.92
50% MAD Flood	0.61	0.07	0.57	0.70	0.84	0.71	0.82	0.98	0.84
Farmers’ Field Replication	1.28	0.29	1.10	0.46	0.67	0.51	0.62	0.90	0.68
Rainfed	1.22	0.44	1.81	0.36	0.38	0.27	0.63	0.67	0.48

### Applied irrigation amount, field water balance, and crop evapotranspiration (ET_C_)

The applied irrigation amount for different irrigation treatments across all seasons is tabulated in Table 7. Compared to the farmers’ field replication, the irrigation amount in the fully irrigated drip treatment increased by 2% during season 1 but decreased by 29.7%, 2.8%, and 2.7% during seasons 2, 3, and 4. For the 50% MAD drip treatment, the irrigation amount decreased by 15.2%, 40.3%, 16.4%, and 16.3% in seasons 1, 2, 3, and 4, respectively. Similarly, for the 50% MAD flood treatment, the irrigation amount decreased by 2.9%, 24.4%, 7.9%, and 7.7% in seasons 1, 2,3, and 4, respectively.

The ET_C_ in this study was estimated as the residual of the field water balance (FWB). The FWB components are tabulated in Table 7 for all the irrigation treatments across four seasons. In Table 7, the term ‘I’ and ‘RF’ represent the total amount of irrigation and rainfall respectively. ‘∆S’ represents the soil moisture storage during the crop season, and ‘DP’ represents deep percolation. Significant differences were observed among the water balance components across all four seasons and the different irrigation treatments. The variations in rainfall and irrigation water application contributed to the variations in soil moisture storage (∆S) throughout the crop seasons. Except for the rainfed treatment, larger values of ∆S were found in season 1 (18.2–40.2 mm), followed by season 3 (18.8–29.5 mm), season 4 (12.8–25.5 mm), and the lowest values were found in season 2 (6.2–13.4 mm). For the rainfed treatment also, the lowest value of ∆S was found in season 2 (14.8 mm) compared to season 1 (52.2 mm), season 3 (56.8 mm), and season 4 (50.8 mm). This was primarily due to the continuous rainfall events in season 2 during the grain-filling stage, which increased soil moisture content at the harvest stage.

**Table 7:** Field water balance (FWB) components for the irrigation treatments across four crop seasons.

Irrigation Treatments	Season 1					Season 2					Season 3					Season 4				
	ET_C_ (mm)	I (mm)	RF (mm)	∆S (mm)	DP (mm)	ET_C_ (mm)	I (mm)	RF (mm)	∆S (mm)	DP (mm)	ET_C_ (mm)	I (mm)	RF (mm)	∆S (mm)	DP (mm)	ET_C_ (mm)	I (mm)	RF (mm)	∆S (mm)	DP (mm)
Fully Irrigated Drip	255.9	210.8	100.5	−34.2	89.6	248.7	123.3	150.2	−11.3	36.1	240.6	213.9	34.3	−19.3	26.9	256.7	216.1	44.9	−13.6	17.9
50% MAD Drip	267.4	175.3	100.5	−30.5	38.9	257.6	104.7	150.2	−13.4	10.7	244.2	183.9	34.3	−30.0	4.0	257.4	185.4	44.9	−32.0	4.9
50% MAD Flood	249.5	200.7	100.5	−18.2	69.9	243.6	132.6	150.2	−6.2	45.4	236.6	202.7	34.3	−18.8	19.2	249.4	204.4	44.9	−12.8	12.7
Farmers’ Field Replication	220.1	206.6	100.5	−30.9	118.0	240.3	175.4	150.2	−7.9	93.2	218.6	220	34.3	−29.5	65.2	221.7	221.5	44.9	−25.5	70.2
Rainfed	151.1	35.4	100.5	−52.2	37.0	169.6	38	150.2	−14.8	33.4	121.5	30.4	34.3	−56.8	0.0	117.7	22	44.9	−50.8	0.0
p-value	< 0.05	< 0.05		< 0.05	< 0.05	< 0.05	< 0.05		< 0.05	< 0.05	< 0.05	< 0.05		< 0.05	< 0.05	< 0.05	< 0.05		< 0.05	< 0.05

(ET_C_: crop evapotranspiration; I and RF: the total amount of water through irrigation and rainfall, respectively; ∆S: soil moisture storage during the crop season; DP: deep percolation; MAD: maximum allowable deficit).

In all the irrigation treatments, the DP was highest in season 1. In this season, heavy rainfall events (52.1 mm and 4.7 mm) during crop development led to high DP in all irrigation treatments. Furthermore, irrigation during the mid-stage coincided with rainfall events, resulting in DP. In season 2, four consecutive rainfall events during the grain-filling stage coincided with the last irrigation, causing DP in all irrigation treatments. In season 3, three rainfall events during the mid-stage occurred after irrigation, resulting in DP. In season 4, the rainfall events during the CRI and grain-filling stages coincided with irrigation, causing DP. In season 1, DP ranged from 38.9 to 118 mm. In season 2, it ranged from 10.7 to 93.2 mm, whereas in season 3, it ranged from 4 to 65.2 mm, and this range was from 4.9 to 70.2 mm in season 4. DP consistently occurred in the farmers’ field replication across all four seasons whenever irrigation was applied. The maximum DP occurred in the farmers’ field replication across all the seasons. The DP in the farmers’ field replication in season 1 was 38% of the total applied water (‘RF + I’), while in season 2, the DP was 29% of the total applied water. Similarly, the DP in seasons 3 and 4 was 26% and 26.3% of the total applied water. The amount of water lost due to deep percolation has been a significant concern in the farmers’ fields in developing countries, resulting in extremely low WUE. The ET_C_ estimated using the FWB was highest for 50% MAD drip, followed by fully irrigated drip, 50% MAD flood, farmers’ field replication, and rainfed treatments across all four seasons. Other studies^81–84^ have also documented differences in water balance components among the irrigation treatments due to variations in rainfall, irrigation quantity, and evapotranspiration.

### Water productivity and water saving strategy

**Fig. 6:**
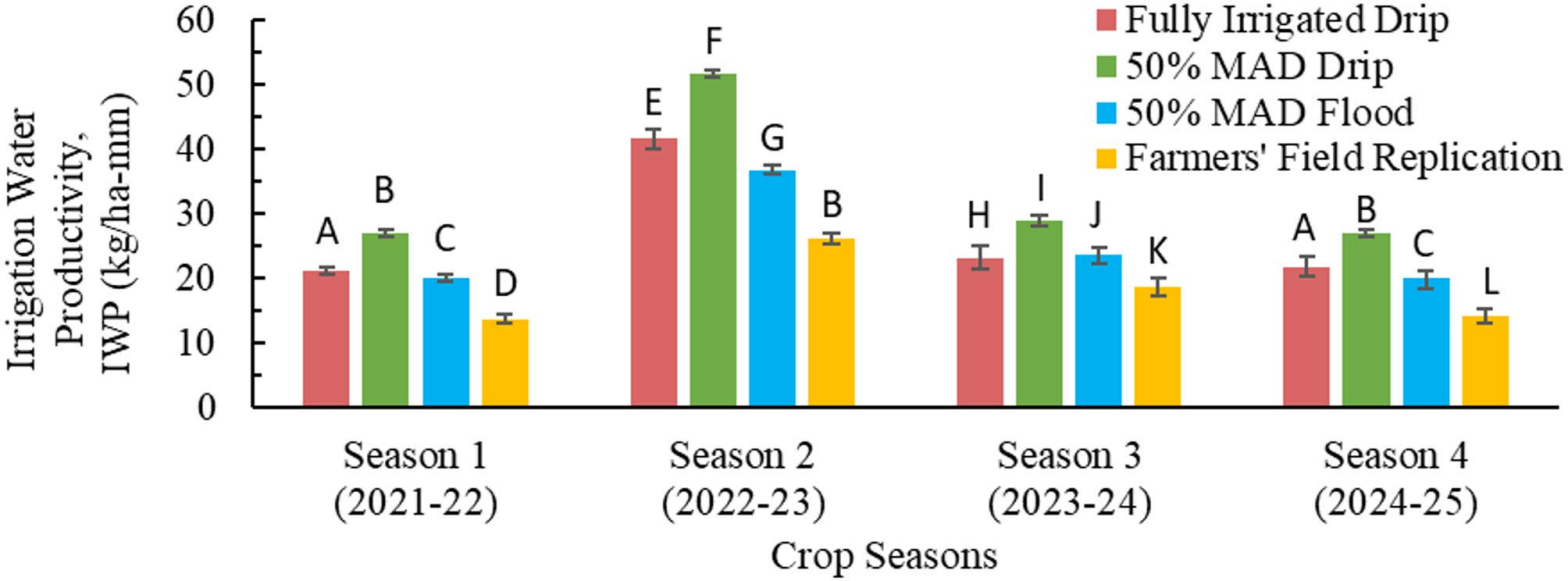
Irrigation water productivity for all irrigation treatments in the four crop seasons. (MAD is maximum allowable deficit; Alphabets in the Figure shows statistical difference; same alphabets show statistical similarity in the values)

The IWP for all irrigation treatments except for the rainfed treatment in all four seasons is shown in Fig. 6. The error bars show the maximum and minimum IWP in each treatment across the seasons. The IWP was highest for 50% MAD drip, followed by fully irrigated drip, 50% MAD flood, and farmers’ field replication in all four seasons. However, the IWP for fully irrigated drip and 50% MAD flood were almost similar in season 3. Further, for all irrigation treatments, the IWP was the highest in season 2. The reason behind the highest IWP during season 2 was the applied irrigation amount, which was lowest for this season (Table 7). Season 2 required less irrigation due to more frequent rainfall than seasons 1, 3, and 4, which reduced the soil moisture deficit and, hence, irrigation requirements. Conversely, in season 1, a heat wave increased the soil moisture deficit, demanding higher irrigation during the flowering and grain-filling stages for fully irrigated drip, 50% MAD drip, and 50% MAD flood irrigation treatments. Similarly, in season 3, high air temperatures during the grain-filling stage exceeded the soil moisture deficit, with minimal rainfall occurring in these seasons. As a result, a greater amount of irrigation was required. Further, compared to season 1, the IWP was greater in season 3, mainly due to reduced grain yield in season 1 caused by a heat wave, despite similar irrigation amounts for a specific treatment between the two seasons. IWP in season 4 was comparable to that in Season 1. This similarity was due to the increase in grain yield in season 4 being nearly proportional to the increase in the amount of irrigation applied. Overall, a consistent trend was observed across all four seasons, indicating that IWP decreased as the irrigation amount increased. Various other studies^24,33,85^ have also reported a reduction in IWP as irrigation rates increased.

**Fig. 7:**
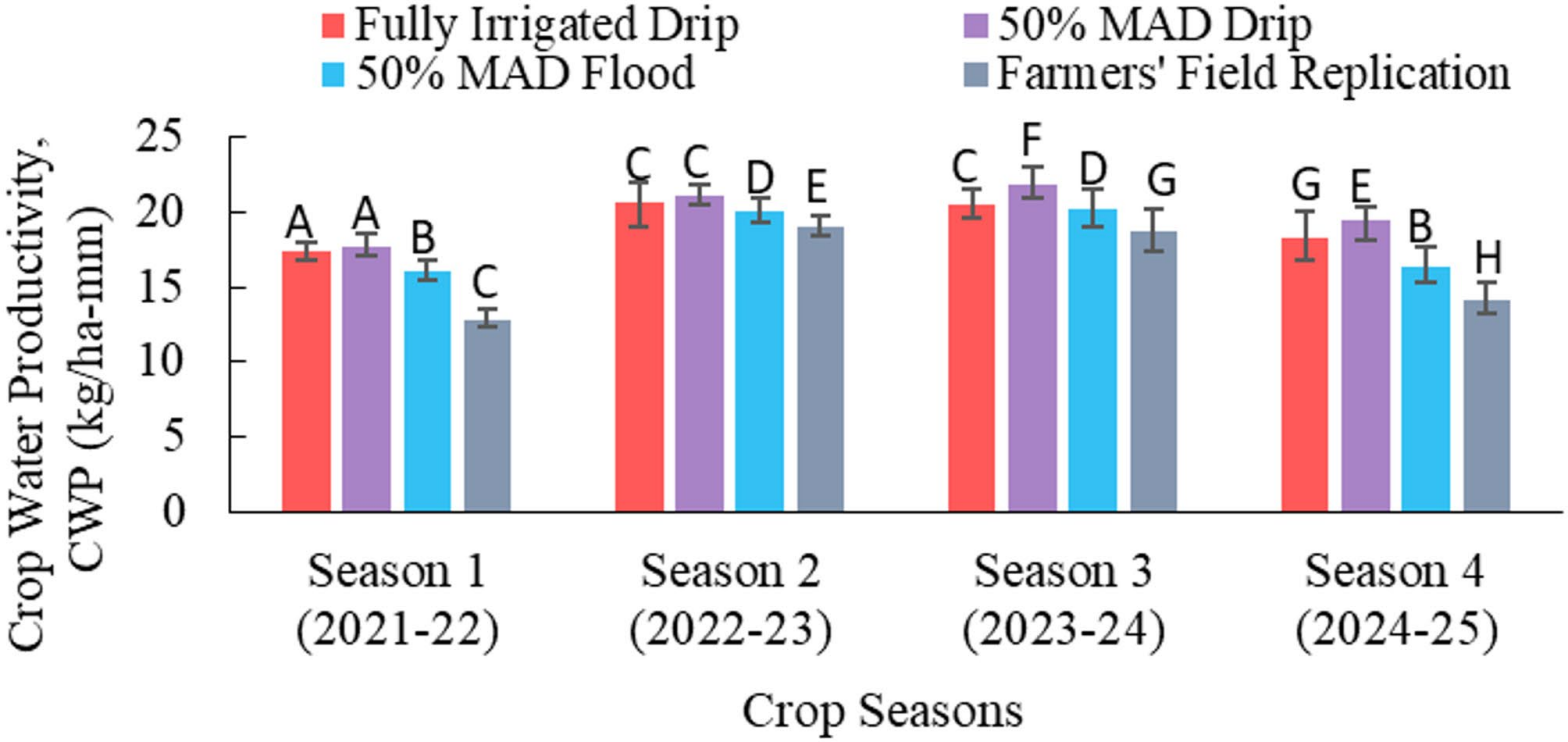
Crop water productivity for all irrigation treatments in the four crop seasons. (MAD is maximum allowable deficit; Alphabets in the Figure shows statistical difference; same alphabets show statistical similarity in the values)

The values of CWP for all irrigation treatments across all four seasons, except for the rainfed treatment, are shown in Fig. 7. The values of CWP in the rainfed treatment across the four seasons were 16.5, 23.3, 21.8, and 16.1 kg ha^− 1^mm^− 1^, respectively. The error bars in Fig. 7 show the maximum and minimum CWP in each treatment across the seasons. In all four seasons, the value of CWP in 50% MAD drip was highest, followed by fully irrigated drip, 50% MAD flood, and the lowest was for farmers’ field replication. The CWP decreased due to a greater grain yield reduction than the crop ET_C_ reduction. CWP indicates the water use efficiency (WUE) of an irrigation treatment. Overall, drip-irrigated treatments exhibited higher WUE compared to flood-irrigated treatments. Other studies^30^ also reported higher WUE in the drip irrigated treatment than in the flood irrigated treatment. Between the two drip irrigated treatments, 50% MAD drip utilized the water more efficiently across the four seasons. The farmers’ field replication exhibited the lowest water use efficiency. Other studies^10,11^ have also reported low water use efficiency in the conventional farmers’ practice in developing countries.

Among all irrigation treatments, the highest GY was observed in 50% MAD drip, followed by fully irrigated drip, 50% MAD flood, and farmers’ field replication across all four seasons. Additionally, the applied irrigation amount was lower in 50% MAD drip, fully irrigated drip, and 50% MAD flood compared to the farmers’ field replication. Further, IWP was highest in 50% MAD drip, followed by fully irrigated drip, 50% MAD flood, and farmers’ field replication across all four seasons. Moreover, water utilization was highest in 50% MAD drip, followed by fully irrigated drip, 50% MAD, and farmers’ field replication across all four seasons, as indicated by CWP. Overall, water saving was highest in 50% MAD drip, fully irrigated drip, and 50% MAD compared to farmers’ field replication without compromising GY.

## Summary, conclusions and challenges

Field experiments were conducted over four consecutive crop seasons (2021–2025) on the wheat crop at the Water Management Field Laboratory, Shiv Nadar Institution of Eminence, Uttar Pradesh, using 25% MAD (maximum allowable deficit) drip, 50% MAD drip, 50% MAD flood, a farmers’ field replication, and rainfed treatments. During the four seasons, the temperature and rainfall patterns varied significantly at critical growth stages. The study aimed to evaluate water-saving strategies by assessing grain yield (GY), irrigation water productivity (IWP), and crop water productivity (CWP) across drip and flood irrigation systems, comparing them to the conventional farmers’ irrigation practices followed in the region. Further, the GY of the irrigation treatments was related to temperature-based heat stress indices. Furthermore, to find out the stable and heat-tolerant irrigation treatments, the heat stress indices based on observed GY were analyzed.

The four seasons’ averaged GY of fully irrigated drip, 50% MAD drip, and 50% MAD flood treatments increased by 31.8%, 40.3%, and 21.4%, respectively, compared to the farmers’ field replication. IWP and CWP were the highest in the 50% MAD drip, and the lowest was in the farmers’ field replication. On average, the CWP in fully irrigated drip, 50% MAD drip, and 50% MAD flood treatments increased by 19.1%, 23.8%, and 12.3%, respectively, compared to the farmers’ field replication. Deep percolation (DP) was significantly higher in the farmers’ field replication than in other treatments across four seasons, averaging 83.1% more than the 50% MAD drip treatment. This substantial DP loss, driven by local thumb rule-based irrigation, contributed to low water use efficiency. Further, the heat vulnerability index was significantly higher in the farmer’s field and rainfed treatments, while their stress tolerance and yield stability indices were comparatively lower than the treatments based on soil moisture observation.

Analysis of GY with heat stress indices based on temperature and observed GY showed that the farmers’ field replication and rainfed treatment were significantly more unstable and vulnerable to the heat wave or stress (in seasons 1 and 3), leading to a notable reduction in GY. Other irrigated treatments, in which irrigation was based on soil moisture observations, were less affected by the heat wave or heat stress induced by interannual variation in weather. The adverse impacts of the varying levels of heat stress induced by interannual variation in weather on wheat yield can be effectively mitigated by employing irrigation scheduling, which was based on soil moisture observations in this study. Findings on GY and water productivity suggest that such practices can be adopted by local farmers to enhance productivity in the backdrop of climate-induced variations. The 50% MAD flood treatment can be adopted by local farmers using their existing flood irrigation systems. However, successful implementation will require the use of soil moisture sensors and adequate technical knowledge of soil moisture-based irrigation scheduling. Furthermore, the flood irrigation system may be replaced with drip irrigation in the future, depending on the financial support made available to local farmers since the system requires a high initial investment for installation. Further, there are various challenges with implementation of drip irrigation system. During the present experiment, several challenges such as emitter damage, filter blockages, and leakage in the drip lines were encountered also encountered.

## Data Availability

The data supporting the findings of this study will be made available by the authors upon reasonable request.
